# Predictors of eHealth Literacy and Its Associations with Preventive Behaviors, Fear of COVID-19, Anxiety, and Depression among Undergraduate Nursing Students: A Cross-Sectional Survey

**DOI:** 10.3390/ijerph19073766

**Published:** 2022-03-22

**Authors:** Ha T. T. Tran, Minh H. Nguyen, Thu T. M. Pham, Giang B. Kim, Hiep T. Nguyen, Ngoc-Minh Nguyen, Hoa T. B. Dam, Thai H. Duong, Yen H. Nguyen, Thao T. Do, Thao T. P. Nguyen, Thuy T. Le, Hien T. T. Do, Tham T. Nguyen, Khue M. Pham, Tuyen Van Duong

**Affiliations:** 1 Faculty of Public Health, Hai Phong University of Medicine and Pharmacy, Hai Phong 042-12, Vietnam; tttha@hpmu.edu.vn (H.T.T.T.); phamminhthu.ytcc@gmail.com (T.T.M.P.); nttham@hpmu.edu.vn (T.T.N.); pmkhue@hpmu.edu.vn (K.M.P.); 2 International Ph.D. Program in Medicine, College of Medicine, Taipei Medical University, Taipei 110-31, Taiwan; d142108015@tmu.edu.tw; 3 School of Public Health, College of Public Health, Taipei Medical University, Taipei 110-31, Taiwan; 4 Institute of Preventive Medicine and Public Health, Hanoi Medical University, Hanoi 115-20, Vietnam; kimbaogiang@hmu.edu.vn; 5 Center for Assessment and Quality Assurance, Hanoi Medical University, Hanoi 115-20, Vietnam; 6 Faculty of Public Health, Pham Ngoc Thach University of Medicine, Ho Chi Minh 725-10, Vietnam; nguyenthanhhiep@pnt.edu.vn (H.T.N.); dr.nguyenngocminh@gmail.com (N.-M.N.); 7 Pham Ngoc Thach Clinic, Pham Ngoc Thach University of Medicine, Ho Chi Minh 725-10, Vietnam; 8 Department of Psychiatry, Thai Nguyen University of Medicine and Pharmacy, Thai Nguyen 241-17, Vietnam; baohoaydtn@gmail.com; 9 Department of Internal Medicine, Thai Nguyen University of Medicine and Pharmacy, Thai Nguyen City 241-17, Vietnam; dhthaivn@gmail.com; 10 Training and Direction of Healthcare Activity Center, Thai Nguyen National Hospital, Thai Nguyen City 241-24, Vietnam; 11 Department of Pharmacology and Clinical Pharmacy, Can Tho University of Medicine and Pharmacy, Can Tho 941-17, Vietnam; nhyen@ctump.edu.vn; 12 Department of Pharmacy, Can Tho University of Medicine and Pharmacy Hospital, Can Tho 941-17, Vietnam; 13 Department of Oral Pathology and Periodontology, Faculty of Odonto-Stomatology, Can Tho University of Medicine and Pharmacy, Can Tho 941-17, Vietnam; dtthao@ctump.edu.vn; 14 Health Personnel Training Institute, Hue University of Medicine and Pharmacy, Thua Thien Hue 491-20, Vietnam; nguyenthiphuongthao@hueuni.edu.vn; 15 Faculty of Medical Laboratory Science, Da Nang University of Medical Technology and Pharmacy, Da Nang 502-06, Vietnam; ltthuy@dhktyduocdn.edu.vn; 16 Faculty of Nursing, Hai Duong Medical Technical University, Hai Duong 031-17, Vietnam; dohienhmtu@gmail.com; 17 School of Nutrition and Health Sciences, Taipei Medical University, Taipei 110-31, Taiwan

**Keywords:** health literacy, preventive behaviors, fear, COVID-19, anxiety, depression, nursing students, handwashing, mask-wearing, physical distancing, Vietnam

## Abstract

Background: The infodemic has been co-existing with the COVID-19 pandemic with an influx of misinformation and conspiracy theories. These affect people’s psychological health and adherence to preventive measures. eHealth literacy (eHEALS) may help with alleviating the negative effects of the infodemic. As nursing students are future healthcare professionals, having adequate eHEALS skills is critically important in their clinical practice, safety, and health. This study aimed to (1) explore the eHEALS level and its associated factors, and (2) examine the associations of eHEALS with preventive behaviors, fear of COVID-19 (FCV-19S), anxiety, and depression among nursing students. Methods: We surveyed 1851 nursing students from 7 April to 31 May 2020 from eight universities across Vietnam. Data were collected, including demographic characteristics, eHEALS, adherence to preventive behaviors (handwashing, mask-wearing, physical distancing), FCV-19S, anxiety, and depression. Linear and logistic regression analyses were performed appropriately to examine associations. Results: The mean score of eHEALS was 31.4 ± 4.4. The eHEALS score was significantly higher in males (unstandardized regression coefficient, B, 0.94; 95% confidence interval, 95% CI, 0.15 to 1.73; *p* = 0.019), and students with a better ability to pay for medication (B, 0.79; 95% CI, 0.39 to 1.19; *p* < 0.001), as compared to their counterparts. Nursing students with a higher eHEALS score had a higher likelihood of adhering to hand-washing (odds ratio, OR, 1.18; 95% CI, 1.15 to 1.22; *p* < 0.001), mask-wearing (OR, 1.15; 95% CI, 1.12 to 1.19; *p* < 0.001), keeping a safe physical distance (OR, 1.20; 95% CI, 1.15 to 1.25; *p* < 0.001), and had a lower anxiety likelihood (OR, 0.95; 95% CI, 0.92 to 0.99; *p* = 0.011). Conclusions: Nursing students who were men and with better ability to pay for medication had higher eHEALS scores. Those with higher eHEALS scores had better adherence to preventive measures, and better psychological health. The development of strategies to improve eHEALS of nursing students may contribute to COVID-19 containment and improve their psychological health.

## 1. Introduction

The advancement of the internet has provided opportunities for people to access information easily from many different fields, especially healthcare [1,2]. With the widespread use of smartphones and computers, people can search for health information, such as disease symptoms, exercise and diet regimens, and illnesses prevention and management advice, at anytime and anywhere [3,4]. However, because of the considerable amount of health information that has been posted on the internet from different sources, the quality of online health information varies [5]. Authoritative health agencies and government institutions often provide evidence-based and higher-quality information than reports or opinions from private blogs or unverified organizations [6].

However, the number of poor health information sources constantly increases on the internet, making it difficult for people to select correct information and make health decisions. Especially during the COVID-19 pandemic, when the spread of the disease worldwide has not been controlled, another public health issue that needs to be addressed is “infodemic”. This term refers to an overwhelming influx of fake or inaccurate information about the epidemic that is circulated on digital networks and other platforms [7,8]. The dissemination of misleading information may make people more confused and worried about the disease and lead to wrong health-related behaviors, hindering governments’ and health agencies’ efforts to contain the disease outbreak [9,10,11]. Therefore, people should have the necessary skills to recognize trusted information sites and the quality of online health information to avoid making wrong health decisions. Those skills are reflected through eHealth literacy (eHEALS).

The eHealth literacy (eHEALS) is defined as “the ability to find, understand and assess health information from electronic platforms and apply acquired knowledge to address health problems” [12]. Improving eHEALS could help people to prevent disease, self-assess, manage their health, and improve health outcomes [13,14]. People with higher eHEALS were more likely to have good health-related knowledge and behaviors, participate in medical screening and healthcare utilization, adhere to physician’s treatment [15,16,17,18,19], which may help to reduce hospitalization rate, healthcare cost, and mortality. However, previous studies also indicated that many people, especially patients, had inadequate eHEALS skills to properly seek, evaluate, and select relevant health information on the internet [20,21,22]. Therefore, patients with low eHEALS need to be educated, and health professionals play a crucial role in supporting those patients to improve eHEALS skills.

Nurses are one of the major labor forces in the healthcare systems, especially in developing countries. In addition, nurses are responsible for communicating with patients and guiding them with medical orders and health-related knowledge. Therefore, healthcare professionals, especially nurses, need to have adequate eHEALS skills to educate patients and their families in effective searching and using online health information. As future health professionals, eHEALS are also crucial for nursing students [23]. Previous research also highlighted the importance of developing the eHEALS skills in future healthcare workers [6]. Good eHEALS skills could help nursing students find and acquire reliable and valid health information to support their study and practice and prepare them with the necessary skills for future work when they become healthcare staff [24]. However, several prior studies showed that nursing students had insufficient skills to assess medical resources and distinguish between low- and high-quality health information on the internet [23,25,26,27]. Therefore, it is important to explore the eHEALS level and its associated factors, which may develop nursing programs to enhance eHealth literacy skills in nursing students.

During the COVID-19 pandemic, nursing students have faced more stress as they had to practice in hospitals and emergency departments, which have high-risk working environments. Thus, strict adherence to preventive behaviors such as wearing a mask, washing hands with soap, or keeping a safe distance with patients is crucial, helping them reduce the risk of infection [28,29]. In addition, nursing students also have an important role in response to the COVID-19 pandemic [30]. In many countries where medical resources are limited and inadequate, medical and nursing students are encouraged to participate in supporting frontline healthcare workers to prevent the spread of COVID-19 [31,32,33].

However, lack of experience, lack of protective equipment, stressful and long-hour work, and fear of infection can affect nursing students’ physical and mental health. Notably, the flood of fake, inaccurate information related to the pandemic on social media and other platforms can cause uncertainty and fear [9], worsening the mental health of nursing students. In addition, many conspiracy theories that were widely spread online, such as anti-vaccination or anti-masks, also had adverse effects on compliance with epidemic prevention measures, jeopardizing public health efforts [34,35]. Thus, enhancing eHEALS could help combat COVID-19 related misinformation on the electronic resources, which may potentially reduce psychological problems and improve adherence to preventive measures [36,37].

Therefore, we conducted an online survey to (1) assess the level of eHEALS and its associated factors; and (2) examine the associations of eHEALS with fear of COVID-19, anxiety, depression, and preventive behaviors among nursing students during the COVID-19 pandemic.

## 2. Methods

### 2.1. Study Design and Sampling

A cross-sectional online survey was carried out on nursing students from 7 April to 31 May 2020. Participants were recruited from eight medical universities across Vietnam, all of which are public universities. This study was approved by the Institutional Review Board of Hanoi University of Public Health in Vietnam (IRB number: 133/2020/YTCC-HD3).

All nursing students from eight universities were informed and encouraged to participate in the survey. Lecturers sent the online survey link to the class leaders, who then shared this link with other students via email, Facebook, or Zalo. Participants signed an online informed consent form before conducting the survey. As all questions were mandatory to answer, there was no missing data. The obtained data was cleaned, coded, and analyzed confidentially.

**Figure 1:**
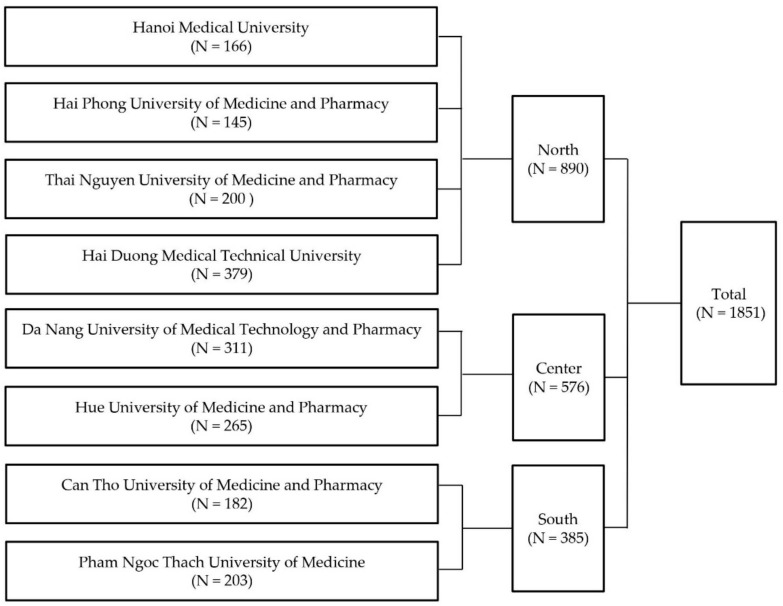
The study sample in different regions.

Out of 3895 possible nursing students, 1851 students completed the survey. In this sample, the margin of error for the eHEALS mean with a 95% confidence level was 0.20 (Text S1 in Supplementary Materials). Figure 1 shows the number of participants at each university.

### 2.2. Measurements

#### 2.2.1. Participant’s Characteristics

We collected data regarding age, gender, academic year (“1–2” vs. “3–4”), ability to pay for medication (“difficult” vs. “easy”). Body Mass Index (BMI, kg/m^2^) was calculated based on self-reported body weight (kg) and height (cm) and classified into three categories: underweight (BMI < 18.5), normal weight (18.5 ≤ BMI < 25.0), and overweight/obese (BMI ≥ 25.0). Students were classified as having COVID-19-like symptoms (Slike-CV19S) if they had any of the following symptoms: fever, cough, difficult breathing, myalgia, fatigue, sputum production, confusion, headache, sore throat, rhinorrhea, chest pain, hemoptysis, diarrhea, and nausea [38]. Student’s comorbidities were assessed using the fourteen items of the Charlson Comorbidity Index [39], and categorized into two groups (“none” vs. “one or more”).

#### 2.2.2. eHealth Literacy

The eight-item eHealth literacy scale was used to evaluate eHEALS in this study. This tool was previously validated in Vietnam [36,40] and was commonly used in nursing students [23,25,27]. The Cronbach’s alpha of this scale in the current study is 0.92. Students were asked to rate how much they agree with eight statements regarding the skills related to seeking, evaluating, and applying health information from electronic resources. Five rating levels range from 1 = “strongly disagree” to 5 = “strongly agree”. The total scores range from 8 to 40, where students with a higher score had better eHEALS.

#### 2.2.3. Fear of COVID-19

The seven-item fear of COVID-19 scale was used to evaluate the level of COVID-19-related fear among nursing students. This scale was validated and widely used on different populations in Vietnam [40,41,42,43]. The Cronbach’s alpha of this tool in our study is 0.87. Students were asked to rate how much they consent with seven opinions about different levels of COVID-19-related fear. Five ranking responses range from 1 = “strongly disagree” to 5 = “strongly agree”. The sum scores range from 7 to 35, where students with a higher score had a higher level of fear.

#### 2.2.4. Anxiety and Depression

This study used the seven-item Generalized Anxiety Disorder (GAD-7) and nine-item Patient Health Questionnaire (PHQ-9) to evaluate anxiety and depression in nursing students. These tools were commonly used to assess psychological problems in Vietnamese studies [44,45,46]. In this study, the Cronbach’s Alpha for PHQ-9 and GAD-7 were 0.89 and 0.92, respectively. Students were asked how much various anxiety and depressive symptoms affected them over the past two weeks. Four response levels include 0 = “not at all”, 1 = “few days”, 2 = “more half of the days”, and 3 = “almost every day”. The total scores of GAD-7 range from 0 to 21, with scores of ≥8 being classified as having anxiety [47]. The sum scores of PHQ-9 are between 0 and 27, with scores of ≥10 being classified as having depression [48].

#### 2.2.5. Preventive Behaviors

This study evaluated preventive behaviors of nursing students with three items, including regular washing hands with soap or alcohol sanitizer, wearing a mask when going outside, and keeping a safe physical distance with others [28]. Students were asked about how often they adhered to the above precautions in the COVID-19 pandemic with five frequency levels, including “never”, “rarely”, “occasionally”, “often”, and “always”. We regrouped each preventive behavior into two categories: “none-adhering” (never, rarely, occasionally, and often) vs. “adhering” (always) [49,50].

### 2.3. Data Analysis

First, the participant’s characteristics and eHEALS were presented as frequency, proportion, mean, standard deviation. Next, we performed the *t*-test and one-way ANOVA test appropriately to compare eHEALS means in different categories of variables. Effect size measures (Cohen’s *d* for the *t*-test or Partial Eta Squared *η*^2^ for the one-way ANOVA) were calculated for between-group difference in eHEALS scores, where Cohen’s d of 0.2, 0.5, and 0.8 or Partial Eta Squared *η*^2^ of 0.01, 0.06, and 0.14 were indicated as small, medium, and large effect sizes, respectively [51]. Then, we used simple and multiple linear regression to examine the influencing factors of eHEALS among nursing students. Finally, the associations of eHEALS with preventive behaviors, fear of COVID-19, anxiety, and depression were tested using adjusted logistic or linear regression (for fear of COVID-19) models. Age, gender, and factors associated with outcome variables at *p*-value < 0.2 in simple regression models were added to the adjusted models (Tables S1 and S2 in Supplementary Materials). To avoid multicollinearity, the Spearman correlation test was conducted to check relationships between adjusted factors. We only added a representative one to adjusted models if two factors were moderately or highly correlated (rho ≥ 0.3) (Table S3 in Supplementary Materials). The *p*-value < 0.05 was defined as a significant level. We analyzed the data using the IBM SPSS Version 26.0 (IBM Corp, Armonk, NY, USA).

## 3. Results

### 3.1. Characteristics of Nursing Students

Most of the sample were female (93.1%) with an average age of 20.5 ± 1.2. Out of 1851 nursing students, 46.0% were third- and fourth-year students, 54.3% found it easy to pay for medication, 4.8% had at least one disease, 21.1% had COVID-19-like symptoms, 2.4% were overweight or obese. The mean score of COVID-19-related fear was 18.7 ± 4.8. The proportions of anxiety and depression among all participants were 6.5% and 11.7%, respectively. Regarding preventive behaviors, 61.4% of participants frequently wore a mask, while only 24.8% and 14.5% nursing students frequently washed hands and practiced physical distancing, respectively. The eHEALS scores were significantly different by categories of gender, ability to pay for medication, COVID-19-like symptoms, preventive behaviors, anxiety, and depression (Table 1).

### 3.2. eHealth Literacy and Associated Factors

Of all 1851 participants, the mean score of eHEALS was 31.4 ± 4.4 (Table 1). The mean scores of each eHEALS item ranged from 3.63 to 4.09. The majority of respondents agreed or strongly agreed that they know what health resources are available (91.3%), where (90.7%) and how (91.5%) to find helpful health resources online, how to use the internet to answer their questions about health (92%) and use online health information to help them (93.6%). However, the high proportions of unsure or disagree or strongly disagree responses were reported in eHEALS skills for evaluating health resources (19.4%), differentiating between high- and low-quality health resources (34.2%), and confidently applying online health information to make health decisions (33.8%) (Table 2).

Table 3 showed the associated factors of eHEALS among using multiple linear regression. Nursing students who had higher eHEALS scores were male (unstandardized regression coefficient, B, 0.94; 95% confident interval, 95% CI, 0.15 to 1.73; *p* = 0.019), those found it easy to pay for medication (B, 0.79; 95% CI, 0.39 to 1.19; *p* < 0.001).

### 3.3. Associations of eHEALS with Preventive Behaviors, Fear of COVID-19, Anxiety, and Depression among Nursing Students

Factors associated with outcome variables at *p*-value < 0.2 were adjusted in final models (Tables S1 and S2 in Supplementary Materials). After adjusting for confounders, the results indicated that nursing students with higher eHEALS had higher likelihoods of high adhering to handwashing (Odds ratio, OR, 1.18; 95% CI, 1.15 to 1.22; *p* < 0.001), mask-wearing (OR, 1.15; 95% CI, 1.12 to 1.19; *p* < 0.001), and keeping a safe physical distance (OR, 1.20; 95% CI, 1.15 to 1.25; *p* < 0.001) (Table 4). We also found that higher eHEALS scores were associated with lower likelihoods of having anxiety disorders (OR, 0.95; 95% CI, 0.92 to 0.99; *p* = 0.011) (Table 5).

## 4. Discussion

In this study, we found that the mean score of eHEALS among nursing students was 31.4. This finding was consistent with previous studies conducted among nursing students in the United States and South Korea [25,52], and was slightly higher than studies in Jordan, Ethiopia, and Sri Lanka [23,53,54]. The eHEALS of nursing students in the current study was also higher than that of other populations, such as adults or college students [15,19,55,56,57]. The inconsistent results between studies and populations may be explained by differences in educational programs, developments in communication technology, and socio-cultural factors in each study location. Although the eHEALS level was relatively high in this study, there were high percentages of nursing students who lacked skills in assessing, differentiating between high- and low-quality health resources, and confidently using online health information to address their health problems. These results were in line with previous studies [23,26,27]. With the rapid development of information technology and smartphones, much health information with different qualities could be easily uploaded to online platforms from various sources. As a result, assessing the quality of online health information and applying it for health decisions is quite tricky, even for nursing students who have better health knowledge. Thus, nursing students need to have adequate eHEALS skills because, as future nurses, they have to assist and educate their patients on evaluating and accessing credible and valid health information to manage and solve health issues. Our results highlighted the poor skills in eHEALS that need to be enhanced among nursing students. Therefore, universities should develop educational curricula that comprehensively improve nursing students’ eHEALS skills, which can benefit their study and future work.

Our study also explored the factors influencing eHEALS levels among nursing students. We found that male students had higher scores of eHEALS than female counterparts. The finding was similar to the results of previous studies among different populations in Taiwan, Ethiopia, and South Korea [53,58,59,60]. The gender difference in eHEALS may be caused by the different routines of using the internet for information searches. In addition, the results indicated that nursing students who found it easy to pay for medication were more like to have better eHEALS. The explanation for this association is that students who can afford medical care may have easier access to health care services. Therefore, they have more opportunities to receive guidance and education in health knowledge and skills from health professionals. Similar findings were also documented in patients and healthcare workers [44,46].

The noticeable results of our study showed that students with a higher eHEALS score had a higher likelihood of adhering to preventive behaviors, including regular washing hands, wearing masking, keeping physical distance. The positive impact of eHEALS on compliance to COVID-19-related protective behaviors has also been documented in prior studies in adults [56,61,62], college students [57,63], and healthcare workers [36]. During the COVID-19 pandemic, the influx of fake news and conspiracy theories are widely spread on social media platforms, raising doubts about the seriousness of COVID-19 and the effectiveness of precaution measures [34,35,64]. People with sufficient eHEALS could evaluate and select accurate and evidence-based sources of information, thereby encouraging them to adhere to appropriate protective behaviors. Our findings are meaningful for nursing students who have to practice in hospitals with high-risk working environments. Therefore, high engagement in preventive behaviors is crucial for nursing students. The current study also examined the association between eHEALS and COVID-19-related fear. Fear is a common feeling when facing danger or threat. Especially, the uncertainty of the COVID-19 pandemic is high with the number of cases and deaths constantly increasing, and there is still no specific COVID-19 treatment [65]. It may explain why there was no relationship between eHEALS and fear of COVID-19 in our study [66]. However, our results showed that students with high eHEALS scores were less likely to have anxiety disorders. This finding was in line with previous studies conducted among adults [17,67]. Enhancing eHEALS can help students avoid unreliable and harmful information from un-verified organizations or commentators [68]. In addition, adequate eHEALS was associated with high compliance with preventive behaviors [62,63], which may help nursing students reduce anxiety about COVID-19 infection. Furthermore, previous studies also showed that eHEALS was positively linked to healthy lifestyles [69,70], such as engaging in healthy diets, staying physically active, which may help them to mitigate the psychological problems.

With a relatively large sample of nursing students, our research can provide reliable evidence to promote nursing training systems to build appropriate teaching strategies that enhance the comprehensive eHEALS skills of nursing students, thereby helping them to improve mental health and adherence to preventive measures during the pandemic. However, several drawbacks should be acknowledged in this study. First, the causal associations could not be drawn from a cross-sectional study. Next, because of a convenience sample, the findings should be generalized for nursing students with caution. Then, given the relatively large sample size and prediction-related research questions, it would be possible to perform powerful analyses (e.g., Path analysis or SEM) to yield more statistically robust data. However, we only used regression analysis to explore potential associations between variables in this study. Therefore, future studies should carry out the statistical method of greater capacity to predict more complex relationships, in order to robust the potential impact of the results. Final, this study did not investigate academic workload that may confound the results. Future studies are required to explore more potential factors and mechanisms.

## 5. Conclusions

In this study, the eHealth literacy score of nursing students was relatively high. However, there were still large percentages of nursing students who lacked skills in assessing and distinguishing between high-quality and low-quality health resources or confidently using online health information to solve health problems. Gender, ability to pay for medication were found to be predictors of eHEALS. Nursing students with a higher eHEALS score had a higher likelihood of compliance to preventive behaviors (handwashing, mask-wearing, physical distancing) and a lower likelihood of having anxiety disorders. Therefore, in order to improve the health of patients and the skill of nursing staff, it is highly required for universities and the health system to integrate eHEALS into training curriculums for nursing students. In addition, potential interventions that enhance nursing students’ eHEALS are also suggested, which may further help improve the adherence to preventive behaviors and mitigate psychological problems during the COVID-19 pandemic.

## Figures and Tables

**Table 1:** Characteristics of nursing students (n = 1851).

Variables	Total (n = 1851)	eHEALS	*p* ^a^	Effect Size ^b^
	n (%)	Mean ± SD		
Age, year, mean ± SD	20.5 ± 1.2			
eHealth literacy, mean ± SD	31.4 ± 4.4			
Gender			0.014	0.23
Female	1723 (93.1)	31.4 ± 4.3		
Male	128 (6.9)	32.4 ± 4.9		
Ability to pay for medication			<0.001	0.19
Very or fairly difficult	846 (45.7)	31.1 ± 4.5		
Very or fairly easy	1005 (54.3)	31.9 ± 4.2		
Academic year			0.728	0.02
1–2	1000 (54.0)	31.4 ± 4.6		
3–4	851 (46.0)	31.5 ± 4.1		
COVID-19-like symptoms			0.059	−0.11
No	1461 (78.9)	31.6 ± 4.4		
Yes	390 (21.1)	31.1 ± 4.3		
Comorbidity			0.307	−0.11
None	1762 (95.2)	31.5 ± 4.4		
One or more	89 (4.8)	31.0 ± 4.5		
BMI, kg/m^2^			0.835	<0.01
Underweight	589 (31.8)	31.5 ± 4.2		
Normal weight	1217 (65.8)	31.4 ± 4.5		
Overweight/obese	44 (2.4)	31.7 ± 3.3		
Handwashing			<0.001	0.58
Non-adhering	1392 (75.2)	30.9 ± 4.4		
Adhering	459 (24.8)	33.3 ± 3.9		
Mask-wearing			<0.001	0.56
Non-adhering	714 (38.6)	30.0 ± 4.6		
Adhering	1137 (61.4)	32.4 ± 4.0		
Physical distancing			<0.001	0.63
Non-adhering	1583 (85.5)	31.1 ± 4.3		
Adhering	268 (14.5)	33.8 ± 4.1		
Depressive symptoms			0.086	−0.12
No (PHQ < 10)	1635 (88.3)	31.5 ± 4.3		
Yes (PHQ ≥ 10)	216 (11.7)	31.0 ± 5.2		
Anxiety Disorder			0.004	−0.27
No (GAD < 8)	1730 (93.5)	31.6 ± 4.3		
Yes (GAD ≥ 8)	121 (6.5)	30.4 ± 5.6		
Fear of COVID-19, mean ± SD	18.7 ± 4.8			

Abbreviation: eHEALS, eHealth literacy; PHQ, Patient Health Questionnaire; GAD, Generalized Anxiety Disorders; SD, standard deviation. ^a^ Results of *t*-test or one-way ANOVA test appropriately. ^b^ Cohen’s *d* for the *t*-test or Partial Eta Squared *η*^2^ for the one-way ANOVA were calculated for between-group difference in eHEALS scores, where Cohen’s d of 0.2, 0.5, and 0.8 or Partial Eta Squared *η*^2^ of 0.01, 0.06, and 0.14 were indicated as small, medium, and large effect sizes, respectively.

**Table 2:** Responses of eHealth literacy scale (n = 1851).

Questions	Strongly Disagree	Disagree	Unsure	Agree	Strongly Agree	Mean ± SD
	n (%)	n (%)	n (%)	n (%)	n (%)	
1. I know what health resources are available on the internet	28 (1.5)	32 (1.7)	102 (5.5)	1382 (74.7)	307 (16.6)	4.03 ± 0.65
2. I know where to find helpful health resources on the internet	26 (1.4)	18 (1.0)	128 (6.9)	1352 (73.0)	327 (17.7)	4.05 ± 0.64
3. I know how to find helpful health resources on the internet	24 (1.3)	17 (0.9)	116 (6.3)	1373 (74.2)	321 (17.3)	4.05 ± 0.62
4. I know how to use the Internet to answer my questions about health	31 (1.7)	14 (0.8)	103 (5.6)	1309 (70.7)	394 (21.3)	4.09 ± 0.66
5. I know how to use the health information I find on the internet to help me	30 (1.6)	14 (0.8)	74 (4.0)	1383 (74.7)	350 (18.9)	4.09 ± 0.63
6. I have the skills I need to evaluate the health resources I find on the internet	27 (1.5)	43 (2.3)	290 (15.7)	1279 (69.1)	212 (11.5)	3.87 ± 0.69
7. I can tell high quality health resources from low quality health resources on the internet	27 (1.5)	82 (4.4)	524 (28.3)	1044 (56.4)	174 (9.4)	3.68 ± 0.76
8. I feel confident in using information from the internet to make health decisions	34 (1.8)	143 (7.7)	448 (24.2)	1067 (57.6)	159 (8.6)	3.63 ± 0.81

**Table 3:** Predictors of eHealth literacy among nursing students (n = 1851).

Variables	Simple Linear Regression		Multiple Linear Regression	
	B (95% CI)	*p*	B (95% CI)	*p*
Age (year), 1-score increment	0.02 (−0.14, 0.17)	0.856	0.04 (−0.12, 0.19)	0.625
Gender				
Female	Ref.		Ref.	
Male	0.99 (0.20, 1.79)	0.014	0.94 (0.15, 1.73)	0.019
Ability to pay for medication				
Very or fairly difficult	Ref.		Ref.	
Very or fairly easy	0.83 (0.42, 1.23)	<0.001	0.79 (0.39, 1.19)	<0.001
Academic year				
1–2	Ref.		-	-
3–4	0.07 (−0.33, 0.47)	0.728	-	-
COVID-19-like symptoms				
No	Ref.		Ref.	
Yes	−0.47 (−0.97, 0.02)	0.059	−0.43 (−0.92, 0.06)	0.086
Comorbidity				
None	Ref.		-	-
One or more	−0.49 (−1.43, 0.45)	0.307	-	-
BMI, kg/m^2^				
Underweight	0.10 (−0.33, 0.53)	0.652	-	-
Normal weight	Ref.		-	-
Overweight/obese	0.30 (−1.03, 1.63)	0.658	-	-

Abbreviation: B, unstandardized regression coefficient; CI, confidence interval.

**Table 4:** Associations of eHealth literacy with preventive behaviors among nursing students (n = 1851).

Variable	Adhering to Handwashing ^a^		Adhering to Mask-Wearing ^b^		Adhering to Physical Distancing ^c^	
	OR (95% CI)	*p*	OR (95% CI)	*p*	OR (95% CI)	*p*
eHealth literacy 1-score increment						
Unadjusted model	1.19 (1.15, 1.22)	<0.001	1.15 (1.12, 1.18)	<0.001	1.20 (1.16, 1.24)	<0.001
Adjusted model	1.18 (1.15, 1.22)	<0.001	1.15 (1.12, 1.19)	<0.001	1.20 (1.15, 1.25)	<0.001

Abbreviations: OR, odds ratio; CI, confidence interval. ^a^ Adjusted for age, gender, ability to pay for medication, COVID-19-like symptoms. ^b^ Adjusted for age, gender, ability to pay for medication, COVID-19-like symptoms, comorbidity, BMI. ^c^ Adjusted for age, gender, ability to pay for medication, COVID-19-like symptoms, comorbidity.

**Table 5:** Associations of eHealth literacy with fear of COVID-19, anxiety, and depression (n = 1851).

Variable	Fear of COVID-19 ^a^		Anxiety ^b^		Depression ^c^	
	B (95% CI)	*p*	OR (95% CI)	*p*	OR (95% CI)	*p*
eHealth literacy 1-score increment						
Unadjusted Model	0.01 (−0.05, 0.05)	0.941	0.95 (0.92, 0.98)	0.004	0.97 (0.94, 1.00)	0.087
Adjusted Model	0.01 (−0.04, 0.06)	0.657	0.95 (0.92, 0.99)	0.011	0.98 (0.95, 1.01)	0.193

Abbreviations: B, unstandardized regression coefficient; OR, odds ratio; CI, confidence interval. ^a^ Adjusted for age, gender, ability to pay for medication, BMI. ^b^ Adjusted for age, gender, ability to pay for medication, COVID-19-like symptoms, comorbidity. ^c^ Adjusted for age, gender, ability to pay for medication, COVID-19-like symptoms, comorbidity, BMI.

## Data Availability

Data will be available on the reasonable request from the corresponding author.

## Supplementary Materials

The following supporting information can be downloaded at: https://www.mdpi.com/article/10.3390/ijerph19073766/s1, Text S1: The margin of error calculation, Table S1: Associated factors of preventive behaviors (n = 1851), Table S2: Associated factors of fear of COVID-19, anxiety, depression, and health-related quality of life (n = 1851), Table S3: Spearman’s correlation (rho) between independent variables among nursing students (n = 1851).

## Abbreviations

B	unstandardized regression coefficient
BMI	Body Mass Index
CI	Confidence interval
eHEALS	eHealth Literacy Scale
FCV-19S	Fear of COVID-19
GAD	Generalized Anxiety Disorder
PHQ	Patient Health Questionnaire
OR	Odds ratio
SD	Standard deviation
