# Supplementary material for: Predictors of eHealth Literacy and Its Associations with Preventive Behaviors, Fear of COVID-19, Anxiety, and Depression among Undergraduate Nursing Students: A Cross-Sectional Survey
Source: Int J Environ Res Public Health. 2022 Mar 22;19(7):3766. doi: 10.3390/ijerph19073766 (PMC8997661; doi:10.3390/ijerph19073766)
Supplement: Supplementary file 1 [file ijerph-19-03766-s001.zip › ijerph-1624539-supplementary.pdf]

Predictors of eHealth Literacy and Its Associations with Preventive Behaviors, Fear of COVID-19, Anxiety, and Depression among Undergraduate Nursing Students: A Cross-sectional Survey

Text S1. The margin of error calculation 2

Table S1. Associated factors of preventive behaviors (n= 1851). 3

Table S2. Associated factors of fear of COVID-19, anxiety, depression (n= 1851). 4

Table S3. Spearman's correlation (rho) between independent variables among nursing students (n= 1851) 5

Text S1. The margin of error calculation

We calculated the margin of error (MOE) of the sample size for the eHEALS mean. Because the population standard deviation (SD) of eHEALS was unknown, the MOE with a 95% confidence interval (CI) was calculated based on the standard error of the sample mean according to a formula:

$$\text{MOE} = 1.96 * \frac{s}{\sqrt{n}} \qquad (1)$$

where 1.96 was the critical value (z-score) for 95% confidence level, $\frac{s}{\sqrt{n}}$ was the standard error of the sample mean with s was the sample SD of eHEALS, and n was the sample size. As a result, the MOE for the eHEALS mean with a 95% CI is 1.96 * $\frac{4.4}{\sqrt{1851}}$ = 0.20. It means that with a 95% confidence level, the sample mean of eHEALS will differ ± 0.2 from the mean of eHEALS in the real population.

Table S1. Associated factors of preventive behaviors (n= 1851).

Variable	Handwashing		Mask-wearing		Physical distancing	
	OR (95% CI)	*p*	OR (95% CI)	*p*	OR (95% CI)	*p*
Age, one-score increment	0.96 (0.89, 1.05)	0.408	0.92 (0.86, 0.99)	0.039	0.81 (0.73, 0.90)	<0.001
Gender						
Female	Ref.		Ref.		Ref.	
Male	0.84 (0.54, 1.29)	0.428	1.05 (0.72, 1.52)	0.796	0.90 (0.53, 1.52)	0.690
Ability to pay for medication						
Very or fairly difficult	Ref.		Ref.		Ref.	
Very or fairly easy	1.36 (1.09, 1.68)	0.005	1.19 (0.99, 1.44)	0.064	1.22 (0.94, 1.58)	0.127
Academic year						
1 – 2	Ref.		Ref.		Ref.	
3 – 4	0.94 (0.76, 1.16)	0.587	0.88 (0.73, 1.06)	0.188	0.65 (0.49, 0.84)	0.001
COVID-19-like symptoms						
No	Ref.		Ref.		Ref.	
Yes	0.76 (0.58, 1.00)	0.053	1.17 (0.93, 1.48)	0.181	0.52 (0.36, 0.76)	0.001
Comorbidity						
No	Ref.		Ref.		Ref.	
Yes	0.71 (0.41, 1.21)	0.204	0.63 (0.41, 0.96)	0.032	0.20 (0.06, 0.63)	0.006
BMI, kg/m^2						
Underweight	0.98 (0.78, 1.23)	0.838	0.83 (0.68, 1.01)	0.063	0.86 (0.64, 1.14)	0.289
Normal weight	Ref.		Ref.		Ref.	
Overweight/obese	0.77 (0.36, 1.62)	0.489	0.93 (0.50, 1.73)	0.831	0.89 (0.37, 2.12)	0.787

Abbreviations: OR, odds ratio; CI, confidence interval.

Table S2. Associated factors of fear of COVID-19, anxiety, depression (n= 1851).

Variable	Fear of COVID-19		Anxiety		Depression	
	B (95% CI)	*p*	OR (95% CI)	*p*	OR (95% CI)	*p*
Age, 1-score increment	-0.16 (-0.33, 0.02)	0.074	0.95 (0.82, 1.10)	0.484	1.02 (0.91, 1.14)	0.776
Gender						
Female	Ref.		Ref.		Ref.	
Male	-0.86 (-1.73, 0.01)	0.051	1.09 (0.54, 2.20)	0.815	1.35 (0.81, 2.25)	0.248
Ability to pay for medication						
1 – 2	Ref.		Ref.		Ref.	
3 – 4	0.74 (0.14, 1.35)	0.015	1.12 (0.69, 1.81)	0.642	0.99 (0.67, 1.46)	0.954
Academic year						
1-2	Ref.		Ref.		Ref.	
3-4	-0.33 (-0.77, 0.11)	0.146	0.79 (0.54, 1.15)	0.212	1.04 (0.78, 1.38)	0.806
COVID-19-like symptoms						
No	Ref.		Ref.		Ref.	
Yes	-0.05 (-0.59, 0.49)	0.852	2.99 (2.04, 4.37)	<0.001	2.76 (2.05, 3.73)	<0.001
Comorbidity						
No	Ref.		Ref.		Ref.	
Yes	-0.48 (-1.51, 0.55)	0.362	2.12 (1.09, 4.09)	0.026	3.41 (2.11, 5.52)	<0.001
BMI, kg/m^2						
Underweight	-0.36 (-0.84, 0.11)	0.132	0.84 (0.55, 1.26)	0.397	0.74 (0.54, 1.03)	0.073
Normal weight	Ref.		Ref.		Ref.	
Overweight/obese	-1.08 (-2.53, 0.37)	0.146	1.37 (0.48, 3.91)	0.561	1.09 (0.46, 2.64)	0.835

Abbreviations: B, unstandardized regression coefficient; OR, odds ratio; CI, confidence interval.

Table S3. Spearman's correlation (rho) between independent variables among nursing students (n= 1851).

Variables	Age	Gender	Academic year	Ability to pay	S-COVID-19-S	Comorbidities
Gender	-.055					
Academic year	**.863**	-.063				
Ability to pay	-.062	.049	-.063			
S-COVID-19-S	-.017	.021	-.007	-.057		
Comorbidities	.031	.058	.046	-.014	.175	
BMI	-.020	.041	.016	-.001	.050	.048

Abbreviation: S-COVID-19-S, COVID-19-like symptoms.
